# Supplementary figures and images for: FBXO7/ntc and USP30 antagonistically set the ubiquitination threshold for basal mitophagy and provide a target for Pink1 phosphorylation in vivo
Source: PLoS Biol. 2023 Aug 3;21(8):e3002244. doi: 10.1371/journal.pbio.3002244 (PMC10427020; doi:10.1371/journal.pbio.3002244)

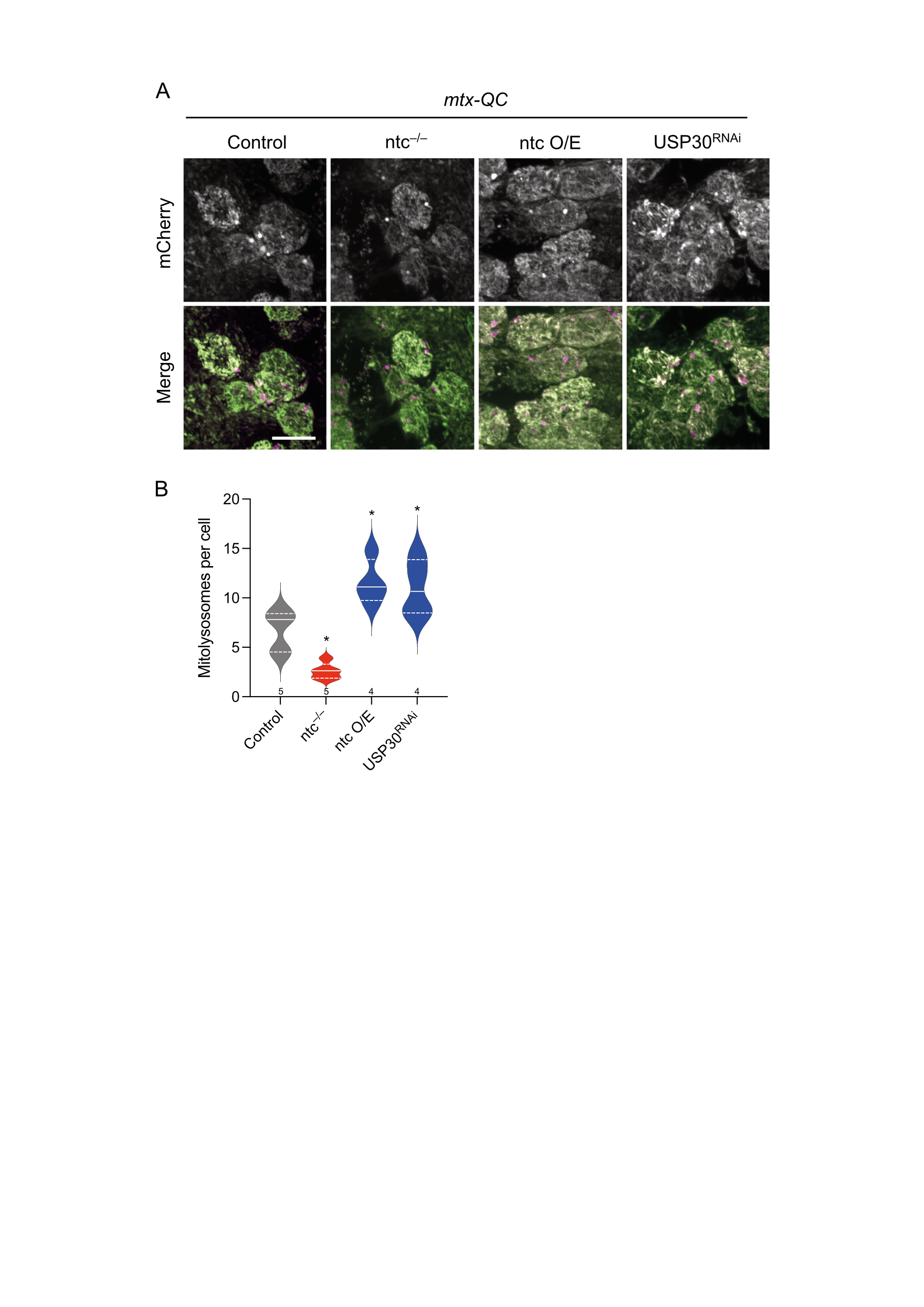

Supplement: S1 Fig — (A, B) Confocal microscopy analysis of the mtx-QC reporter in larval CNS of control, ntc mutant, ntc overexpression and USP30 knockdown with the pan-neuronal driver nSyb-GAL4. Mitolysosomes are evident as GFP-negative/mCherry-positive (red-only) puncta; n shown in chart. One-way ANOVA with Bonferroni post hoc test correction; * P < 0.05. Scale bars = 10 μm. Full details of numerical data and analyses underlying the quantitative data can be found in S1 Data. (TIFF) [file pbio.3002244.s001.tiff]

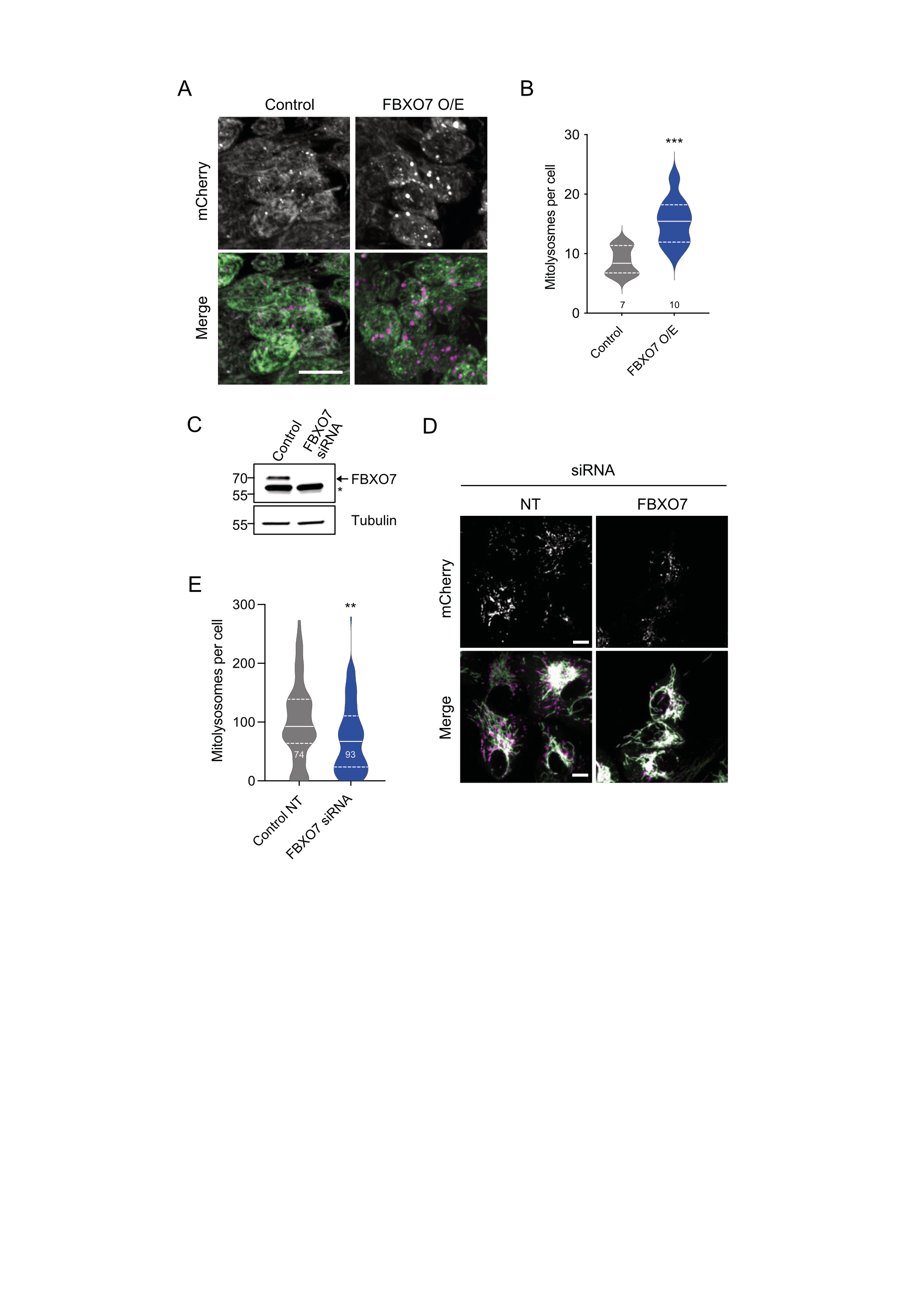

Supplement: S2 Fig — (A, B) Confocal microscopy analysis of the mito-QC reporter in larval CNS of control and the transgenic expression of FBXO7 with the pan-neuronal driver nSyb-GAL4. Mitolysosomes are evident as GFP-negative/mCherry-positive (red-only) puncta; n shown in chart. One-way ANOVA with Bonferroni post hoc test correction; *** P < 0.001. Scale bar = 10 μm. (C) Immunoblot analysis of the knockdown of FBXO7 in human ARPE-19 cells expressing the mito-QC reporter. Arrow shows FBXO7 band; * shows nonspecific band. (D, E) Confocal microscopy analysis of the mito-QC reporter in ARPE-19 human cell line of control siRNA and FBXO7 siRNA. Mitolysosomes are evident as GFP-negative/mCherry-positive (red-only) puncta; n shown in chart. Two-tailed t test; ** P < 0.01. Scale bars = 10 μm. Full details of numerical data and analyses underlying the quantitative data can be found in S1 Data. (TIFF) [file pbio.3002244.s002.tiff]

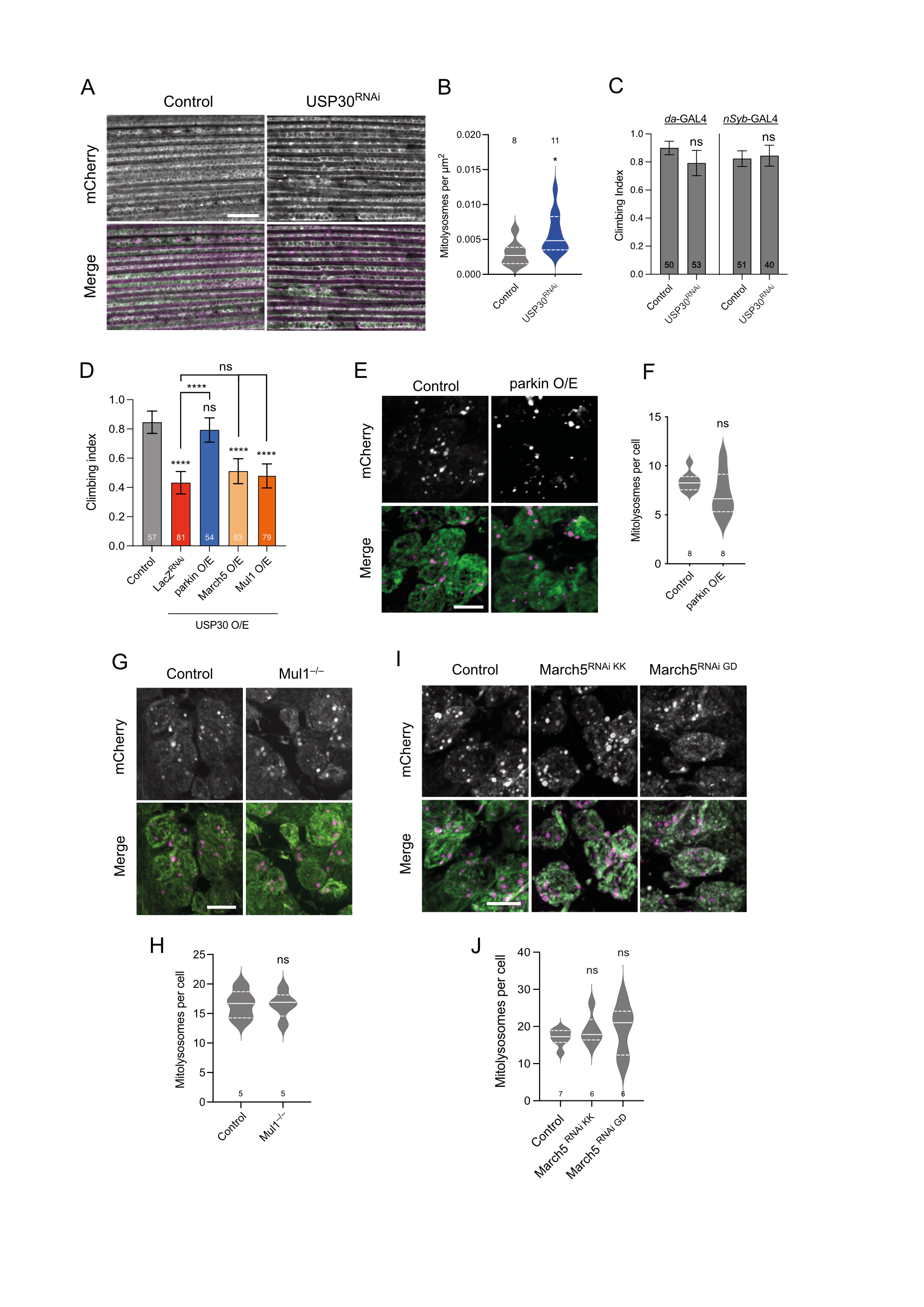

Supplement: S3 Fig — (A, B) Confocal microscopy analysis of the mito-QC reporter in 2-day-old adult thoraces of control and USP30 knockdown with the muscular driver Mef2-GAL4. Mitolysosomes are evident as GFP-negative/mCherry-positive (red-only) puncta; n shown in chart. Two-tailed t test; * P < 0.05. Scale bar = 10 μm. (C) Climbing ability of 2-day-old flies expressing USP30 RNAi with the ubiquitous driver da-GAL4 or with the pan-neuronal driver nSyb-GAL4. Chart show mean ± 95% CI and n values. Kruskal–Wallis nonparametric test with Dunn’s post hoc test correction for multiple comparisons. (D) Climbing ability of 10-day-old flies overexpressing USP30 alone or in combination with parkin, March5 or Mul1 with the ubiquitous driver Act-GAL4. Chart show mean ± 95% CI and n values. Kruskal–Wallis nonparametric test with Dunn’s post hoc test correction for multiple comparisons; **** P < 0.0001. (E, F) Confocal microscopy analysis of the mito-QC reporter in larval CNS of control and parkin overexpression with the pan-neuronal driver nSyb-GAL4. Mitolysosomes are evident as GFP-negative/mCherry-positive (red-only) puncta; n shown in chart. Two-tailed t test. Scale bar = 10 μm. (G, H) Confocal microscopy analysis of the mito-QC reporter in larval CNS of control and Mul1 mutant. Mitolysosomes are evident as GFP-negative/mCherry-positive (red-only) puncta; n shown in chart. Two-tailed t test. Scale bar = 10 μm. (I, J) Confocal microscopy analysis of the mito-QC reporter in larval CNS of control and March5 knockdowns with the pan-neuronal driver nSyb-GAL4. Mitolysosomes are evident as GFP-negative/mCherry-positive (red-only) puncta; n shown in chart. Two-tailed t test. Scale bar = 10 μm. Full details of numerical data and analyses underlying the quantitative data can be found in S1 Data. (TIFF) [file pbio.3002244.s003.tiff]

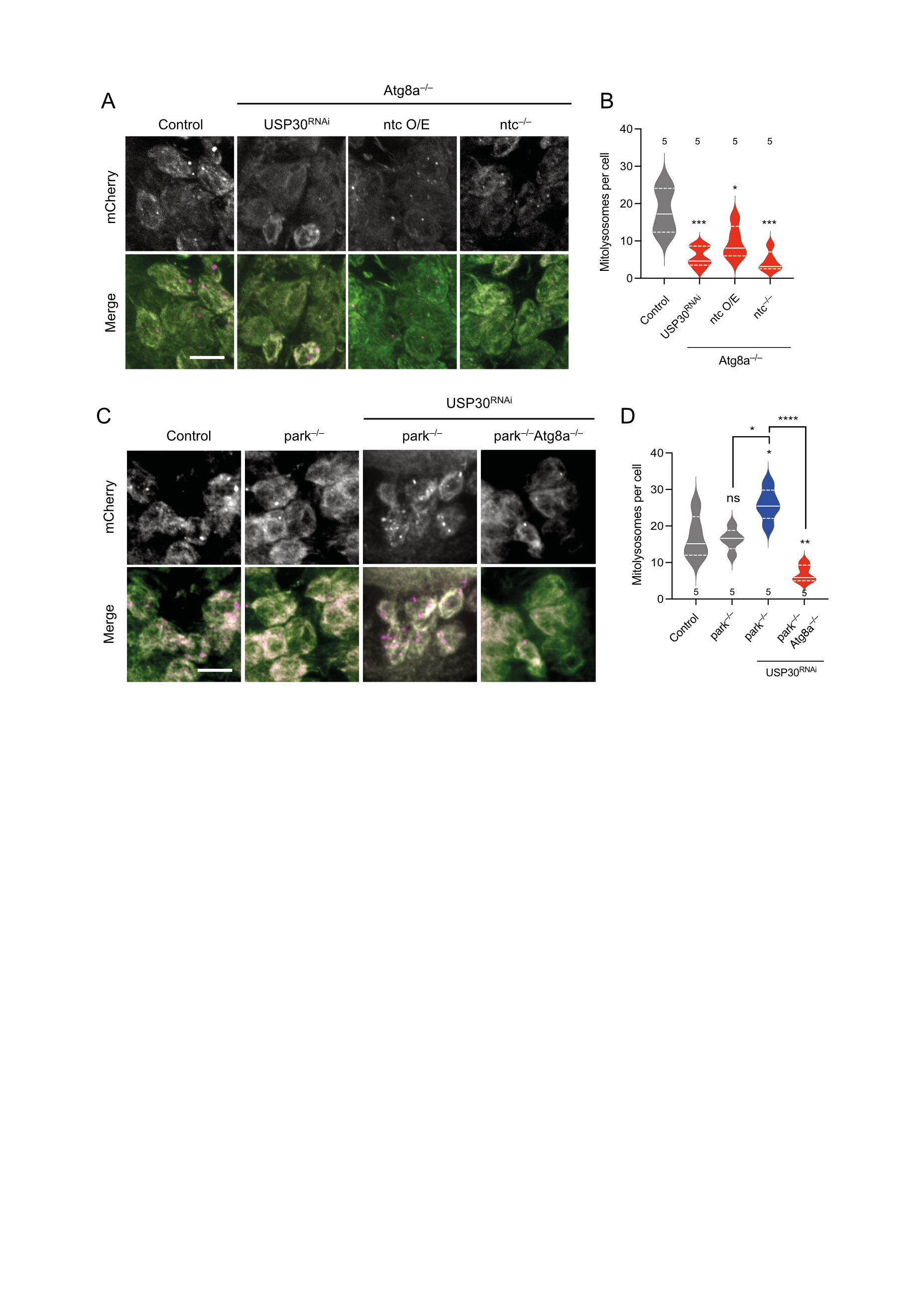

Supplement: S4 Fig — (A, B) Confocal microscopy analysis of the mito-QC reporter in larval CNS of control, knockdown of USP30, overexpression of ntc, and ntc mutant in the Atg8a mutant background with the pan-neuronal driver nSyb-GAL4. Mitolysosomes are evident as GFP-negative/mCherry-positive (red-only) puncta; n shown in chart. One-way ANOVA with Bonferroni post hoc test correction; * P < 0.05, *** P < 0.001. Scale bar = 10 μm. (C, D) Confocal microscopy analysis of the mito-QC reporter in larval CNS of control and parkin mutant alone or with the USP30 knockdown in the presence or absence of Atg8a, with the pan-neuronal driver nSyb-GAL4. Mitolysosomes are evident as GFP-negative/mCherry-positive (red-only) puncta; n shown in chart. One-way ANOVA with Bonferroni post hoc test correction; * P < 0.05, ** P < 0.01, **** P < 0.0001. Scale bar = 10 μm. Full details of numerical data and analyses underlying the quantitative data can be found in S1 Data. (TIFF) [file pbio.3002244.s004.tiff]

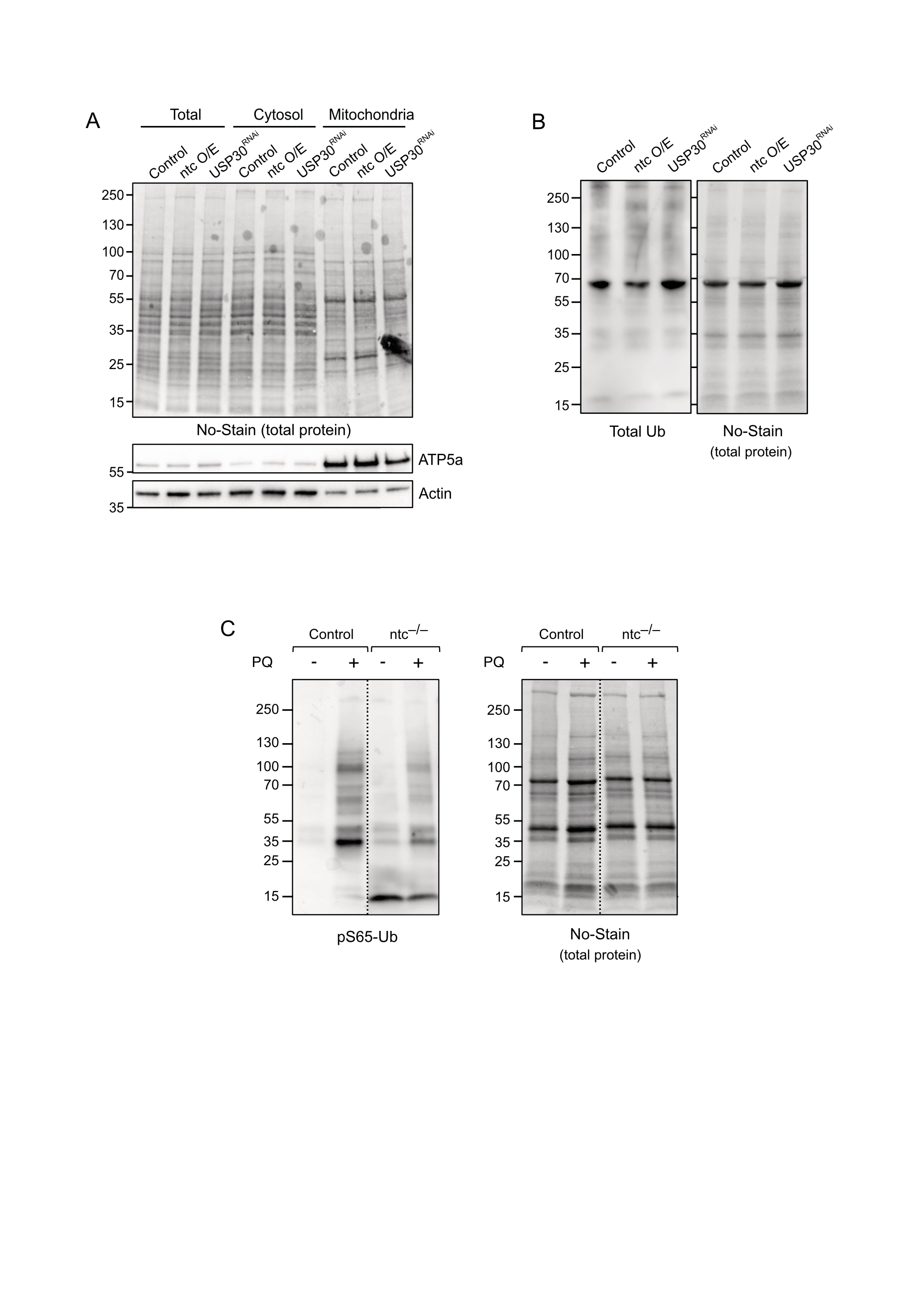

Supplement: S5 Fig — (A) Representative immunoblot of the subcellular fractionation of 2-day-old flies with the transgenic expression of ntc or USP30 RNAi with the ubiquitous driver da-GAL4. Cytosolic- and mitochondria-enriched fractions are label with Actin and ATP5a, respectively. (B) Representative immunoblot of total ubiquitin (FK2) of 2-day-old flies in control, ntc overexpression and USP30 knockdown with the ubiquitous driver da-GAL4. (C) Representative immunoblot of pS65-Ub levels of 2-day-old flies treated with paraquat (PQ) in control and ntc mutants. (TIFF) [file pbio.3002244.s005.tiff]
