# Supplementary material for: FBXO7/ntc and USP30 antagonistically set the ubiquitination threshold for basal mitophagy and provide a target for Pink1 phosphorylation in vivo
Source: PLoS Biol. 2023 Aug 3;21(8):e3002244. doi: 10.1371/journal.pbio.3002244 (PMC10427020; doi:10.1371/journal.pbio.3002244)
Supplement: S1 Table — (DOCX) [file pbio.3002244.s006.docx]

**Supplementary Table 1**. Details of full genotypes used in this study. More details of each line can be found in Methods.

| **Figure 1** | |
| --- | --- |
| **Label** | **Genotype** |
| **A** | |
| Control | UAS-LacZ/+; da-GAL4/+ |
| park^–/–^ | UAS-LacZ/+; da-GAL4, park^25^/park^25^ |
| park^–/–^; ntc O/E | UAS-ntc/+; da-GAL4, park^25^/park^25^ |
| **B** | |
| Control | UAS-LacZ/+; da-GAL4/+ |
| park^–/–^ | UAS-LacZ/+; da-GAL4, park^25^/park^25^ |
| park^–/–^; ntc O/E | UAS-ntc/+; da-GAL4, park^25^/park^25^ |
| **C** | |
| Control | UAS-LacZ/+; da-GAL4/+ |
| park^–/–^ | UAS-LacZ/+; da-GAL4, park^25^/park^25^ |
| park^–/–^; ntc | UAS-ntc/+; da-GAL4, park^25^/park^25^ |
| **D** | |
| Control | UAS-LacZ/+; da-GAL4/+ |
| park^–/–^ | UAS-LacZ/+; da-GAL4, park^25^/park^25^ |
| park^–/–^; ntc O/E | UAS-ntc/+; da-GAL4, park^25^/park^25^ |
| **E** | |
| Control | UAS-LacZ/+; da-GAL4/+ |
| park^–/–^ | UAS-LacZ/+; da-GAL4, park^25^/park^25^ |
| park^–/–^; ntc O/E | UAS-ntc/+; da-GAL4, park^25^/park^25^ |
| **F** | |
| Control | UAS-LacZ/+; da-GAL4/+ |
| Pink1^–^ | Pink1^B9^/Y; UAS-LacZ/+; da-GAL4/+ |
| Pink1^–^; ntc O/E | Pink1^B9^/Y; UAS-ntc/+; da-GAL4/+ |
| **G** | |
| Control | UAS-LacZ/+; da-GAL4/+ |
| Pink1^–^ | Pink1^B9^/Y; UAS-LacZ/+; da-GAL4/+ |
| Pink1^–^; ntc O/E | Pink1^B9^/Y; UAS-ntc/+; da-GAL4/+ |
| **H** | |
| Control | UAS-LacZ/+; da-GAL4/+ |
| Pink1^–^ | Pink1^B9^/Y; UAS-LacZ/+; da-GAL4/+ |
| Pink1^–^; ntc O/E | Pink1^B9^/Y; UAS-ntc/+; da-GAL4/+ |
| **I** | |
| Control | GMR-GAL4/UAS-LacZ |
| ntc O/E | GMR-GAL4, UAS-LacZ/UAS-ntc |
| Pink1 O/E | GMR-GAL4, UAS-Pink1/UAS-LacZ |
| Pink1+ntc O/E | GMR-GAL4, UAS-Pink1/UAS-ntc |

| **Figure 2** | |
| --- | --- |
| **Label** | **Genotype** |
| **A** | |
| Control | UAS-LacZ/+; da-GAL4/+ |
| ntc^–/–^ | ntc^ms771^/ntc^ms771^ |
| ntc^–/Df^ | ntc^ms771^/Df(3L)Exel6097 |
| ntc^–/–^, ntc^rescue^ | UAS-ntc; da-GAL4, ntc^ms771^/ ntc^ms771^ |
| **B** | |
| Control | UAS-LacZ/+; da-GAL4/+ |
| ntc^–/–^ | ntc^ms771^/ntc^ms771^ |
| ntc^–/Df^ | ntc^ms771^/Df(3L)Exel6097 |
| ntc^–/–^, ntc^rescue^ | UAS-ntc; da-GAL4, ntc^ms771^/ ntc^ms771^ |
| **C** | |
| Control | w^1118^ |
| ntc^–/–^ | ntc^ms771^/ntc^ms771^ |
| ntc^–/–^, ntc^rescue^ | UAS-ntc; da-GAL4, ntc^ms771^/ntc^ms771^ |
| **D** | |
| Control | w^1118^ |
| ntc^–/–^ | ntc^ms771^/ntc^ms771^ |
| **E** | |
| Control | UAS-LacZ/+; da-GAL4/+ |
| ntc^–/–^ | ntc^ms771^/ntc^ms771^ |
| ntc^–/Df^ | ntc^ms771^/Df(3L)Exel6097 |
| **F** | |
| Control | w^1118^ |
| ntc^–/–^ | ntc^ms771^/ntc^ms771^ |
| **G** | |
| Control | w^1118^ |
| ntc^–/–^ | ntc^ms771^/ntc^ms771^ |
| **H** | |
| Control | w^1118^ |
| ntc^–/–^ | ntc^ms771^/ntc^ms771^ |
| ntc^–/–^, ntc^rescue^ | UAS-ntc; da-GAL4, ntc^ms771^/ntc^ms771^ |
| **I** | |
| Control | w^1118^ |
| ntc^–/–^ | ntc^ms771^/ntc^ms771^ |

| **Figure 3** | |
| --- | --- |
| **Label** | **Genotype** |
| **A** | |
| Control | mito-QC/UAS-LacZ^RNAi^; nSyb-GAL4/+ |
| ntc^–/–^ | mito-QC/UAS-LacZ^RNAi^; nSyb-GAL4, ntc^ms771^/ntc^ms771^ |
| ntc^–/–^, ntc^rescue^ | mito-QC/UAS-ntc; nSyb-GAL4, ntc^ms771^/ntc^ms771^ |
| **B** | |
| Control | mito-QC/UAS-LacZ^RNAi^; nSyb-GAL4/+ |
| ntc^–/–^ | mito-QC/UAS-LacZ^RNAi^; nSyb-GAL4, ntc^ms771^/ntc^ms771^ |
| ntc^–/–^, ntc^rescue^ | mito-QC/UAS-ntc; nSyb-GAL4, ntc^ms771^/ntc^ms771^ |
| **C** | |
| Control | mito-QC/UAS-LacZ^RNAi^; nSyb-GAL4/+ |
| ntc O/E | mito-QC/UAS-ntc; nSyb-GAL4/+ |
| **D** | |
| Control | mito-QC/UAS-LacZ^RNAi^; nSyb-GAL4/+ |
| ntc O/E | mito-QC/UAS-ntc; nSyb-GAL4/+ |
| **E** | |
| Control | mito-QC/UAS-LacZ^RNAi^; nSyb-GAL4/+ |
| USP30^RNAi^ | mito-QC/UAS-USP30^RNAi^; nSyb-GAL4/+ |
| USP30^RNAi^, ntc^–/–^ | mito-QC/UAS-USP30^RNAi^; nSyb-GAL4, ntc^ms771^/ntc^ms771^ |
| **F** | |
| Control | mito-QC/UAS-LacZ^RNAi^; nSyb-GAL4/+ |
| USP30^RNAi^ | mito-QC/UAS-USP30^RNAi^; nSyb-GAL4/+ |
| USP30^RNAi^, ntc^–/–^ | mito-QC/UAS-USP30^RNAi^; nSyb-GAL4, ntc^ms771^/ntc^ms771^ |
| **G** | |
| Control | Act-GAL4/UAS-LacZ |
| USP30 O/E + LacZ | Act-GAL4, UAS-USP30/UAS-LacZ |
| USP30 O/E + ntc O/E | Act-GAL4, UAS-USP30/UAS-ntc |

| **Figure 4** | |
| --- | --- |
| **Label** | **Genotype** |
| **A** | |
| Control | mito-QC/UAS-LacZ^RNAi^; nSyb-GAL4/+ |
| park^–/–^ | mito-QC/UAS-LacZ^RNAi^; nSyb-GAL4, park^25^/park^25^ |
| park^–/–^, ntc O/E | mito-QC/UAS-ntc; nSyb-GAL4, park^25^/park^25^ |
| **B** | |
| Control | mito-QC/UAS-LacZ^RNAi^; nSyb-GAL4/+ |
| park^–/–^ | mito-QC/UAS-LacZ^RNAi^; nSyb-GAL4, park^25^/park^25^ |
| park^–/–^, ntc O/E | mito-QC/UAS-ntc; nSyb-GAL4, park^25^/park^25^ |
| **C** | |
| Control | mito-QC/UAS-LacZ^RNAi^; nSyb-GAL4/+ |
| Pink1^–^ | Pink1^B9^/Y; mito-QC/UAS-LacZ^RNAi^; nSyb-GAL4/+ |
| Pink1^–^, ntc O/E | Pink1^B9^/Y; mito-QC/UAS-ntc; nSyb-GAL4/+ |
| **D** | |
| Control | mito-QC/UAS-LacZ^RNAi^; nSyb-GAL4/+ |
| Pink1^–^ | Pink1^B9^/Y; mito-QC/UAS-LacZ^RNAi^; nSyb-GAL4/+ |
| Pink1^–^, ntc O/E | Pink1^B9^/Y; mito-QC/UAS-ntc; nSyb-GAL4/+ |

| **Figure 5** | |
| --- | --- |
| **Label** | **Genotype** |
| **A** | |
| Control | mito-QC/UAS-LacZ^RNAi^; nSyb-GAL4/+ |
| park^–/–^ | mito-QC/UAS-LacZ^RNAi^; nSyb-GAL4, park^25^/park^25^ |
| park^–/–^, USP30^RNAi^ | mito-QC/UAS-USP30^RNAi^; nSyb-GAL4, park^25^/park^25^ |
| park^–/–^, USP30^RNAi^, ntc^–/–^ | mito-QC/UAS-USP30^RNAi^; nSyb-GAL4, park^25^, PBac{WH}CG10855^f07259^/ntc^ms771^ |
| **B** | |
| Control | mito-QC/UAS-LacZ^RNAi^; nSyb-GAL4/+ |
| park^–/–^ | mito-QC/UAS-LacZ^RNAi^; nSyb-GAL4, park^25^/park^25^ |
| park^–/–^, USP30^RNAi^ | mito-QC/UAS-USP30^RNAi^; nSyb-GAL4, park^25^/park^25^ |
| park^–/–^, USP30^RNAi^, ntc^–/–^ | mito-QC/UAS-USP30^RNAi^; nSyb-GAL4, park^25^, PBac{WH}CG10855^f07259^/ntc^ms771^ |
| **C** | |
| Control | mito-QC/ UAS-LacZ^RNAi^; nSyb-GAL4/+ |
| Pink1^–^ | Pink1^B9^/Y; mito-QC/UAS-LacZ^RNAi^; nSyb-GAL4/+ |
| Pink1^–^, USP30^RNAi^ | Pink1^B9^/Y; mito-QC/UAS-USP30^RNAi^; nSyb-GAL4/+ |
| Pink1^–^, USP30^RNAi^, ntc^–/–^ | Pink1^B9^/Y; mito-QC/UAS-USP30^RNAi^; nSyb-GAL4, ntc^ms771^/ntc^ms771^ |
| **D** | |
| Control | mito-QC/ UAS-LacZ^RNAi^; nSyb-GAL4/+ |
| Pink1^–^ | Pink1^B9^/Y; mito-QC/UAS-LacZ^RNAi^; nSyb-GAL4/+ |
| Pink1^–^, USP30^RNAi^ | Pink1^B9^/Y; mito-QC/UAS-USP30^RNAi^; nSyb-GAL4/+ |
| Pink1^–^, USP30^RNAi^, ntc^–/–^ | Pink1^B9^/Y; mito-QC/UAS-USP30^RNAi^; nSyb-GAL4, ntc^ms771^/ntc^ms771^ |

| **Figure 6** | |
| --- | --- |
| **Label** | **Genotype** |
| **A** | |
| Control | UAS-LacZ/+; da-GAL4/+ |
| USP30^RNAi^ | UAS-USP30^RNAi^/+; da-GAL4/+ |
| ntc O/E | UAS-ntc/+; da-GAL4/+ |
| ntc^–/–^ | ntc^ms771^/ntc^ms771^ |
| **B** | |
| Control | UAS-LacZ/+; da-GAL4/+ |
| USP30^RNAi^ | UAS-USP30^RNAi^/+; da-GAL4/+ |
| ntc O/E | UAS-ntc/+; da-GAL4/+ |
| ntc^–/–^ | ntc^ms771^/ntc^ms771^ |
| **C** | |
| Control | UAS-LacZ/+; da-GAL4/+ |
| USP30^RNAi^ | UAS-USP30^RNAi^/+; da-GAL4/+ |
| ntc O/E | UAS-ntc/+; da-GAL4/+ |
| ntc^–/–^ | ntc^ms771^/ntc^ms771^ |
| **D** | |
| Control | UAS-LacZ/+; da-GAL4/+ |
| USP30^RNAi^ | UAS-USP30^RNAi^/+; da-GAL4/+ |
| ntc O/E | UAS-ntc/+; da-GAL4/+ |
| ntc^–/–^ | ntc^ms771^/ntc^ms771^ |
| **E** | |
| Control | UAS-GFP-mCherry-Atg8a/UAS-LacZ^RNAi^/+; nSyb-GAL4/+ |
| USP30^RNAi^ | UAS-GFP-mCherry-Atg8a/UAS-USP30^RNAi^/+; nSyb-GAL4/+ |
| ntc O/E | UAS-GFP-mCherry-Atg8a/UAS-ntc/+; nSyb-GAL4/+ |
| ntc^–/–^ | UAS-GFP-mCherry-Atg8a/UAS-LacZ^RNAi^/+; nSyb-GAL4, ntc^ms771^/ntc^ms771^ |
| **F** | |
| Control | UAS-GFP-mCherry-Atg8a/UAS-LacZ^RNAi^/+; nSyb-GAL4/+ |
| USP30^RNAi^ | UAS-GFP-mCherry-Atg8a/UAS-USP30^RNAi^/+; nSyb-GAL4/+ |
| ntc O/E | UAS-GFP-mCherry-Atg8a/UAS-ntc/+; nSyb-GAL4/+ |
| ntc^–/–^ | UAS-GFP-mCherry-Atg8a/UAS-LacZ^RNAi^/+; nSyb-GAL4, ntc^ms771^/ntc^ms771^ |

| **Figure 7** | |
| --- | --- |
| **Label** | **Genotype** |
| **A** | |
| Control | UAS-LacZ/+; da-GAL4/+ |
| ntc O/E | UAS-ntc/+; da-GAL4/+ |
| USP30^RNAi^ | UAS-USP30^RNAi^/+; da-GAL4/+ |
| **B** | |
| Control | UAS-LacZ/+; da-GAL4/+ |
| ntc O/E | UAS-ntc/+; da-GAL4/+ |
| USP30^RNAi^ | UAS-USP30^RNAi^/+; da-GAL4/+ |
| **C** | |
| Control | w^1118^ |
| park^–/–^ | park^25^/park^25^ |
| ntc^–/–^ | ntc^ms771^/PBac{WH}CG10855^f07259^ |
| park^–/–^, ntc^–/–^ | park^25^, ntc^ms771^/park^25^, PBac{WH}CG10855^f07259^ |
| **D** | |
| Control | w^1118^ |
| park^–/–^ | park^25^/park^25^ |
| ntc^–/–^ | ntc^ms771^/PBac{WH}CG10855^f07259^ |
| park^–/–^, ntc^–/–^ | park^25^, ntc^ms771^/park^25^, PBac{WH}CG10855^f07259^ |
| **E** | |
| Control | UAS-LacZ/+; da-GAL4/+ |
| park^–/–^ | UAS-LacZ/+; da-GAL4, park^25^/park^25^ |
| park^–/–^; ntc O/E | UAS-ntc/+; da-GAL4, park^25^/park^25^ |
| **F** | |
| Control | UAS-LacZ/+; da-GAL4/+ |
| park^–/–^ | UAS-LacZ/+; da-GAL4, park^25^/park^25^ |
| park^–/–^; ntc O/E | UAS-ntc/+; da-GAL4, park^25^/park^25^ |

| **Supplementary Figure 1** | |
| --- | --- |
| **Label** | **Genotype** |
| **A** | |
| Control | mtx-QC/UAS-LacZ^RNAi^; nSyb-GAL4/+ |
| USP30^RNAi^ | mtx-QC/UAS-USP30^RNAi^; nSyb-GAL4/+ |
| ntc | mtx-QC/UAS-ntc; nSyb-GAL4/+ |
| ntc^–/–^ | mtx-QC/UAS-LacZ^RNAi^; nSyb-GAL4, ntc^ms771^/ntc^ms771^ |
| **B** | |
| Control | mtx-QC/UAS-LacZ^RNAi^; nSyb-GAL4/+ |
| USP30^RNAi^ | mtx-QC/UAS-USP30^RNAi^; nSyb-GAL4/+ |
| ntc | mtx-QC/UAS-ntc; nSyb-GAL4/+ |
| ntc^–/–^ | mtx-QC/UAS-LacZ^RNAi^; nSyb-GAL4, ntc^ms771^/ntc^ms771^ |

| **Supplementary Figure 2** | |
| --- | --- |
| **Label** | **Genotype** |
| **A** | |
| Control | mito-QC/UAS-LacZ^RNAi^; nSyb-GAL4/+ |
| FBXO7 O/E | mito-QC/UAS-FBXO7; nSyb-GAL4/+ |
| **B** | |
| Control | mito-QC/UAS-LacZ^RNAi^; nSyb-GAL4/+ |
| FBXO7 O/E | mito-QC/UAS-FBXO7; nSyb-GAL4/+ |

| **Supplementary Figure 3** | |
| --- | --- |
| **Label** | **Genotype** |
| **A** | |
| Control | Mef2-GAL4/UAS-LacZ^RNAi^; mito-QC/+ |
| USP30^RNAi^ | Mef2-GAL4/UAS-USP30^RNAi^; mito-QC/+ |
| **B** | |
| Control | Mef2-GAL4/UAS-LacZ^RNAi^; mito-QC/+ |
| USP30^RNAi^ | Mef2-GAL4/UAS-USP30^RNAi^; mito-QC/+ |
| **C** | |
| Control | UAS-LacZ^RNAi^/+; da-GAL4/+ |
| USP30^RNAi^ | UAS-USP30^RNAi^/+; da-GAL4/+ |
| Control | UAS-LacZ^RNAi^/+; nSyb-GAL4/+ |
| USP30^RNAi^ | UAS-USP30^RNAi^/+; nSyb-GAL4/+ |
| **D** | |
| Control | Act-GAL4/UAS-LacZ |
| USP30 O/E + LacZ | Act-GAL4, UAS-USP30/UAS-LacZ |
| USP30 O/E + parkin O/E | Act-GAL4, UAS-USP30/UAS-parkin |
| USP30 O/E + March5 O/E | Act-GAL4, UAS-USP30/+; UAS-March5/+ |
| USP30 O/E + Mul1 O/E | Act-GAL4, UAS-USP30/+; UAS-Mul1/+ |
| **E** | |
| Control | mito-QC/UAS-LacZ^RNAi^; nSyb-GAL4/+ |
| parkin O/E | mito-QC/UAS- parkin_C2_; nSyb-GAL4/+ |
| **F** | |
| Control | mito-QC/UAS-LacZ^RNAi^; nSyb-GAL4/+ |
| parkin O/E | mito-QC/UAS- parkin_C2_; nSyb-GAL4/+ |
| **G** | |
| Control | mito-QC/+; nSyb-GAL4/+ |
| Mul1^–/–^ | mito-QC/+; nSyb-GAL4, Mul1[A6]/Mul1[A6] |
| **H** | |
| Control | mito-QC/+; nSyb-GAL4/+ |
| Mul1^–/–^ | mito-QC/+; nSyb-GAL4, Mul1[A6]/Mul1[A6] |
| **I** | |
| Control | mito-QC/UAS-LacZ^RNAi^; nSyb-GAL4/+ |
| March5^RNAi KK^ | mito-QC/UAS-March5^RNAi KK^; nSyb-GAL4/+ |
| March5^RNAi GD^ | mito-QC/UAS-March5^RNAi GD^; nSyb-GAL4/+ |
| **J** | |
| Control | mito-QC/UAS-LacZ^RNAi^; nSyb-GAL4/+ |
| March5^RNAi KK^ | mito-QC/UAS-March5^RNAi KK^; nSyb-GAL4/+ |
| March5^RNAi GD^ | mito-QC/UAS-March5^RNAi GD^; nSyb-GAL4/+ |

| **Supplementary Figure 4** | |
| --- | --- |
| **Label** | **Genotype** |
| **A** | |
| Control | mito-QC/UAS-LacZ^RNAi^; nSyb-GAL4/+ |
| Atg8a^–/–^, USP30^RNAi^ | Atg8a[KG07569]/Y; mito-QC/UAS-USP30^RNAi^; nSyb-GAL4/+ |
| Atg8a^–/–^, ntc O/E | Atg8a[KG07569]/Y; mito-QC/UAS-ntc; nSyb-GAL4/+ |
| Atg8a^–/–^, ntc^–/–^ | Atg8a[KG07569]/Y; mito-QC/ LacZ^RNAi^; nSyb-GAL4, ntc^ms771^/ntc^ms771^ |
| **B** | |
| Control | mito-QC/UAS-LacZ^RNAi^; nSyb-GAL4/+ |
| Atg8a^–/–^, USP30^RNAi^ | Atg8a[KG07569]/Y; mito-QC/UAS-USP30^RNAi^; nSyb-GAL4/+ |
| Atg8a^–/–^, ntc O/E | Atg8a[KG07569]/Y; mito-QC/UAS-ntc; nSyb-GAL4/+ |
| Atg8a^–/–^, ntc^–/–^ | Atg8a[KG07569]/Y; mito-QC/ LacZ^RNAi^; nSyb-GAL4, ntc^ms771^/ntc^ms771^ |
| **C** | |
| Control | mito-QC/UAS-LacZ^RNAi^; nSyb-GAL4/+ |
| park^–/–^ | mito-QC/UAS-LacZ^RNAi^; nSyb-GAL4, park^25^/park^25^ |
| USP30^RNAi^, park^–/–^ | mito-QC/UAS-USP30^RNAi^; nSyb-GAL4, park^25^/park^25^ |
| Atg8a^–/–^, USP30^RNAi^, park^–/–^ | Atg8a[KG07569]/Y; mito-QC/UAS-USP30^RNAi^; nSyb-GAL4, park^25^/park^25^ |
| **D** | |
| Control | mito-QC/UAS-LacZ^RNAi^; nSyb-GAL4/+ |
| park^–/–^ | mito-QC/UAS-LacZ^RNAi^; nSyb-GAL4, park^25^/park^25^ |
| USP30^RNAi^, park^–/–^ | mito-QC/UAS-USP30^RNAi^; nSyb-GAL4, park^25^/park^25^ |
| Atg8a^–/–^, USP30^RNAi^, park^–/–^ | Atg8a[KG07569]/Y; mito-QC/UAS-USP30^RNAi^; nSyb-GAL4, park^25^/park^25^ |

| **Supplementary Figure 5** | |
| --- | --- |
| **Label** | **Genotype** |
| **A** | |
| Control | UAS-LacZ/+; da-GAL4/+ |
| ntc O/E | UAS-ntc/+; da-GAL4/+ |
| USP30^RNAi^ | UAS-USP30^RNAi^/+; da-GAL4/+ |
| **B** | |
| Control | UAS-LacZ/+; da-GAL4/+ |
| ntc O/E | UAS-ntc/+; da-GAL4/+ |
| USP30^RNAi^ | UAS-USP30^RNAi^/+; da-GAL4/+ |
| **C** | |
| Control | w^1118^ |
| ntc^–/–^ | ntc^ms771^/ntc^ms771^ |
