# Supplementary material for: FBXO7/ntc and USP30 antagonistically set the ubiquitination threshold for basal mitophagy and provide a target for Pink1 phosphorylation in vivo
Source: PLoS Biol. 2023 Aug 3;21(8):e3002244. doi: 10.1371/journal.pbio.3002244 (PMC10427020; doi:10.1371/journal.pbio.3002244)
Supplement: S1 Raw Images — (PDF) [file pbio.3002244.s008.pdf]

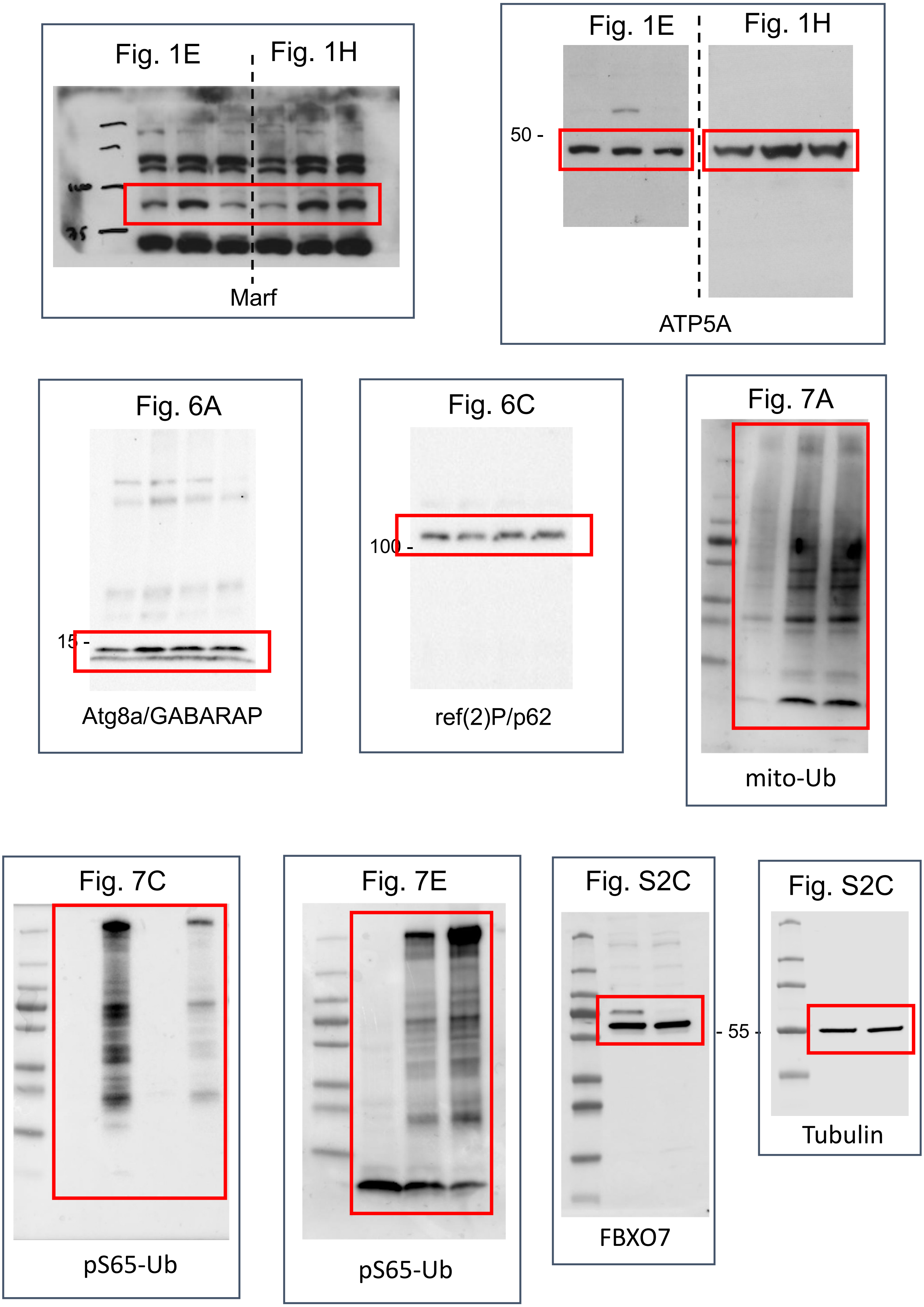
Fig. 1E
Fig. 1H
Marf
Fig. 1E
Fig. 1H
50 -
ATP5A
Fig. 6A
15 -
Atg8a/GABARAP
Fig. 6C
100 -
ref(2)P/p62
Fig. 7A
mito-Ub
Fig. 7C
pS65-Ub
Fig. 7E
pS65-Ub
Fig. S2C
FBXO7
- 55 -
Fig. S2C
Tubulin

Fig. S5A

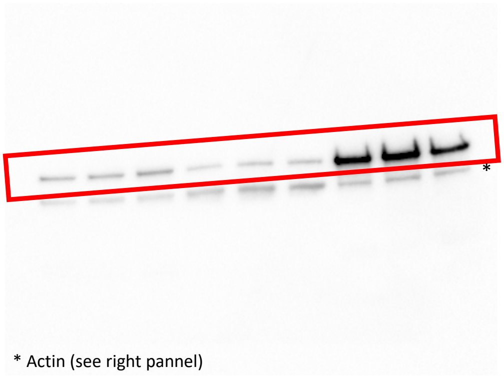

ATP5a

Fig. S5A

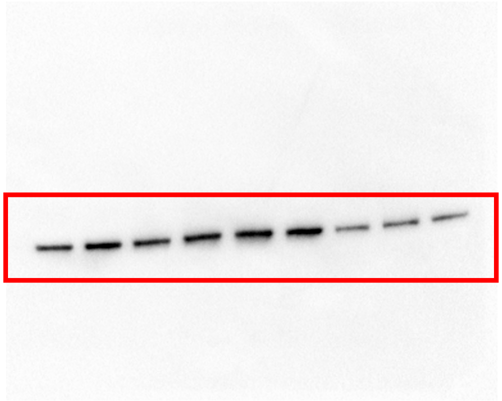

Actin

Fig. S5B

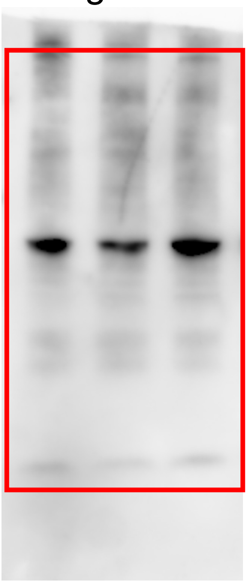

Total Ub

Fig. S5C

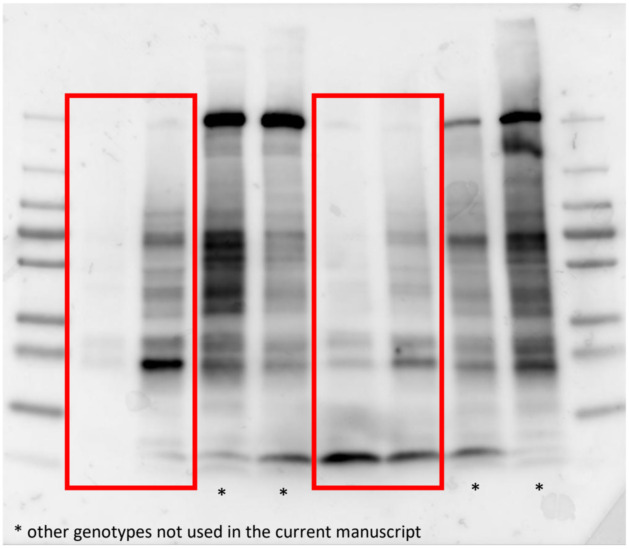

pS65-Ub
