# Supplementary material for: FBXO7/ntc and USP30 antagonistically set the ubiquitination threshold for basal mitophagy and provide a target for Pink1 phosphorylation in vivo
Source: PLoS Biol. 2023 Aug 3;21(8):e3002244. doi: 10.1371/journal.pbio.3002244 (PMC10427020; doi:10.1371/journal.pbio.3002244)
Supplement: Attachment — Submitted filename: 132243_1_rebuttal_2639823_rsdj4j.pdf [file pbio.3002244.s009.pdf]

Full Revision

Review COMMONS

Manuscript number: RC-2022-01769R
Corresponding author(s): Alvaro Sanchez-Martinez, Alexander J. Whitworth

1. General Statements [optional]

We thank all the reviewers for their time and effort in the peer-review process. We appreciate the positive reflections on the study and the feedback comments which were well thought-out and articulated. Considering these comments has led us to deeper reflections on the conceptualization of the mechanisms at play, and we hope that our responses here and revisions of the manuscript have improved the presentation of the data and our interpretation of these complex matters. As a result, we have now incorporated a new supplementary figure 5 and present a new model figure with the corresponding comments in the text.

2. Point-by-point description of the revisions

Reviewer #1 (Evidence, reproducibility and clarity (Required)):

In this manuscript Sanchez-Martinez et al investigated the function of ntc, a Drosophila homologue of FBXO7. The mechanisms by which mutations in this protein cause autosomal recessive PD are poorly understood. The protein has previously been implicated in PINK1/Parkin mitophagy however the mechanistic detail is lacking. The data presented here provide an important insight into the molecular functions of ntc as well as mitophagy in vivo in general. Ntc was found to promote ubiquitination of mitochondrial proteins which is counteracted by USP30. The basal ubiquitination regulated by these two enzymes is proposed to act as a permissive factor for the initiation of Pink1/Parkin mitophagy. The conclusions are based on strong data in vivo and there is a lot to like about this paper. The analyses are done rigorously, conclusions are balanced and well supported and there is a lot of conceptual novelty in the dataset. At the same time the paper raises some questions with regard to the role of mitophagy, at least in Drosophila. Not all of these could be answered during revisions but it would be useful to address the points outlined below.

1. The functional measurements such as climbing, flight and lifespan are used to complement the data on mitophagy and mitochondrial health. However, it is clear that these do not correlate. Ntc KOs and Pink1/Parkin flies have reduced climbing and flight ability, however ntc KO does not affect mitochondrial function. In case of Pink1/Parkin the assumption is that impaired fly functionality is due to damaged mitochondria. This is clearly not the case with Ntc. How relevant is climbing/flight/lifespan to the role of Ntc in mitophagy?

- The reviewer raises a very good point, and we agree that there isn't a strict, linear connection between the cellular process of mitophagy and the presentation of organismal neuromuscular phenotypes such as motor behaviours and lifespan. Considering this point further, starts to highlight the complexity of the situation at hand: it is becoming clear that there are many different forms of mitophagy, and these perform different functions in cellular remodelling and homeostasis. And, of course, there are many ways to interfere with neuromuscular function (as well as lifespan). So, it follows that some forms of mitophagy may dramatically impact neuromuscular homeostasis when disrupted, while others may not. We and others have described that basal mitophagy is minimally by loss of *Pink1/parkin* in vivo, so the organismal phenotypes clearly do not relate to this biology. But it is currently unclear how the phenotypes may relate to physiologically relevant stress-induced mitophagy as the precise nature of this, as well as the methods to experimentally manipulate it, are lacking.

Here, we are initially documenting new phenotypes for ntc, with no bias for the mechanistic cause, all of which are worthy of description to gain a holistic view of the overall contribution of this gene function to organismal integrity. It is clear from the literature that ntc/FBXO7 has multiple functions, for instance, regulating proteasome function and caspase activation, so it follows that genetic loss is likely to impinge on multiple cellular functions causing pleiotropic effects.

We have always been careful not to consider (or claim) that the organismal phenotypes, such as motor function or lifespan, are specifically due to defective mitophagy but are an overall readout of the health and functioning of the neuromuscular system. Nevertheless, these phenotypes are useful in investigating manipulations that improve or worsen the effect of *Pink1/parkin* (or *ntc*) mutants, which, *a priori*, may or may not also modulate mitophagy. While we have documented these new organismal phenotypes of *ntc* mutants and analysed the impact on basal and stress-induced mitophagy, we have not drawn a cause-effect link between the two but a correlation at least. Nevertheless, this issue raises some important considerations for the field in terms of the different kinds of mitophagy, and when they may be needed, and the impact on cells, systems or whole-organisms when they are defective. This issue is explored more in answer to Q3 below.

2. Fig 3 is somewhat patchy, mitophagy is shown for USP30 and ntc KO epistasis but climbing index used in OE setting. These data do not match, and it feels like experiments that are shown are the ones that worked. The relevance of climbing index to mitophagy is unclear as mentioned above. Does KO/OE of ntc and USP30 affect levels of mitochondria, e.g. Marf used as a maker for mitochondria in Fig. 1? And if not why not, considering that ntc/USP30 but not PINK/Parkin control basal mitophagy?

- The main purpose of the data in Figure 3 was to document the impact of ntc manipulation on basal mitophagy, and by extension to link this to the known mitophagy regulator, USP30, whose loss of function has been documented to promote stress-induced mitophagy. Here, we successfully demonstrated *USP30* RNAi and *ntc* OE cause an increase in mitophagy, and established their genetic relationship. However, it is very important to our *modus operandi* that we have orthogonal evidence for this relationship and understand the impact at an organismal level. As the reviewer indicates, the obvious choice that would align with the mitophagy data would

be to assess whether loss of *ntc* prevents a *USP30* RNAi phenotype. However, in our hands *USP30* knockdown using the same RNAi line had no discernible impact on viability or behaviour in adult flies, precluding this experiment. Of note, we did observe a detrimental impact of *USP30* knockdown on adult viability using a different RNAi line (KK) but this has 2 known off-targets so this result is unreliable. An alternative approach to genetically test the antagonistic relationship between USP30 and ntc, and equally valid in our view, is to assess whether *ntc* OE can counteract a *USP30* OE phenotype. Here, we were fortunate that *USP30* OE does indeed provoke an organismal phenotype, and this was suppressed by *ntc* OE consistent with the mitophagy data. It is unfortunate that the more obvious option was not workable on this occasion, but we hold that the genetic relationship was nevertheless substantiated as expected, albeit with alternative manipulations. Importantly, we established the validity of this approach by demonstrating the known genetic interaction between USP30 and parkin, whereby USP30 OE locomotor phenotype is suppressed by parkin OE (now, Fig. S3D). To substantiate this approach more clearly, we have now added to the text (lines 200-201) and figure (Fig. S3C) the lack of observable effect by *USP30* knockdown as noted above.

As to the second point, assessing whether levels of mitochondria are changed by ntc/USP30 manipulations; according to the immunoblot presented in new Supplementary Figure 5A (and replicates), the levels of ATP5a are not notably changed by ntc O/E or USP30 RNAi. Marf levels are also unchanged though this is not shown. This is in line with our expectations since, as discussed above, ntc/USP30 are only one set of regulators of one type of mitophagy and several others exist. The reviewer will likely be aware that the levels of mitochondria are tightly regulated and fine-tuned for the specific need in different tissue types, and that substantial changes to mitochondrial content can be catastrophic for cell and tissue viability. While this is relatively straightforward to achieve in cultured cells, substantial reductions in mitochondrial content are non-viable in an *in vivo* context. Of course, in physiological conditions, rates of degradation are kept in fine-balance with biogenesis and proliferation so non-catastrophic alterations in mitochondrial content are usually counteracted by compensatory changes in proliferation or degradation.

3. What is the role of mitophagy in the maintenance of mitochondrial function in Drosophila in general? Pink1/Parkin KO assumed to result in dysfunctional mitochondria due to impairment of damage-induced mitophagy which is a minor contributor to mitophagy as has previously been published by the authors and confirmed in this dataset using mitophagy reporter. At the same time ntc is clearly required for mitophagy, but mitochondria remains structurally and functionally intact in ntc KO. The most straightforward interpretation of these data is that Pink1/Parkin contribute little to mitophagy in flies and their effect on mitochondria and fly function is independent of mitophagy. Instead ntc (and USP30) strongly regulate mitophagy but mitophagy is not important for the maintenance of mitochondrial function. The effect of ntc on fly function is also independent of its role in mitophagy/mitochondria. Unless there is an alternative explanation the entire dataset would need to be reinterpreted and discussed differently.

- We agree that this is an important point raised by the study findings and needs to be clearly articulated in the text, but we don’t think it is as simple as whether ‘mitophagy’ contributes to

mitochondrial and organismal integrity. First, as mentioned above, it is becoming apparent that it is crucial for the field to clearly and consciously distinguish between basal and induced forms of mitophagy. Basal mitophagy is likely, though not yet proven, to be an important component of mitochondrial quality control in metazoans and largely act in a house-keeping manner providing continual surveillance of mitochondrial quality and quantity. As such, like many other critical biological processes, it is likely to be supported in a 'belt-and-braces' manner by several mechanisms working in parallel with a degree of functional redundancy. In contrast, induced mitophagy is presumed to be quiescent until stimulated into action at specific times for specific purposes. For instance, it is assumed, though not yet proven, that PINK1/Parkin stress-induced mitophagy is stimulated in response to some kind of physiological stress or damage to mitochondria that may be catastrophic if left unchecked.

We and others have shown before that PINK1/Parkin are minimally involved in basal mitophagy in vivo but they are well-established to promote stress-induced mitophagy. In contrast, we have found that ntc regulates basal mitophagy and, we posit, facilitates PINK1/Parkin mitophagy by providing the initiating ubiquitination. How does this map onto the mitochondrial/organismal phenotypes? There are clear disruptions to energy-intensive, mitochondria-rich tissues in *Pink1/parkin* mutants which are not grossly affected in *ntc* mutants. On the other hand, *ntc* mutants show a dramatically short lifespan, much shorter than *Pink1/parkin* mutants, while other measures of mitochondrial integrity are fine.

The *Pink1/parkin* phenotypes are consistent with a catastrophic loss of tissue integrity caused by the lack of a crucial protective measure (induced mitophagy) for a specific circumstance (we think, mitochondrial 'damage' arising from a huge metabolic burst). In contrast, while loss of *ntc* causes a partial (but not complete) loss of basal mitophagy, these same tissues appear to be able to cope with this impact on house-keeping QC but importantly are also able to mount a stress-induced response via Pink1/parkin still being present. On the other hand, it should be remembered that ntc is known to perform other important cellular functions, such as regulating proteasome function and caspase activation, and it is perhaps loss of these functions that causes the dramatic loss of vitality.

Importantly, although Pink1/parkin do not contribute to basal (steady-state) mitophagy, we think it is not appropriate to think of Pink1/parkin mitophagy as a 'minor' contribution. Since, under the particular triggering conditions of damage or stress that stimulate Pink/parkin mitophagy, apparently only Pink1/parkin can perform this role in certain *Drosophila* tissues, and this stress-induced mitophagy is crucial to tissue integrity, as exemplify by the fact that increasing basal mitophagy via ntc O/E still is not sufficient to rescue Pink1 mutants. In this specific context, this is a *major* mitophagy pathway.

In summary, the connection between mitochondrial autophagic degradation and mitochondrial/organismal health is not a simple one and we would avoid conflating different aspects of mitochondrial QC with the expectation that the consequences of their dysfunction would be the same. Nevertheless, these well-considered feedback comments have crystallised

the need to elaborate these ideas in the Discussion where we have added a new section (lines 359-387).

Reviewer #1 (Significance (Required)):

Very strong genetic data presented; novel functions for human Park15 homologue in Drosophila; mechanistic insight into the ubiquitination of mitochondria by two opposing enzymes. Overall very interesting paper but interpretation is less clear which needs to be addressed.

Reviewer #2 (Evidence, reproducibility and clarity (Required)):

In this ms. Sanchez-Martinez and colleagues study the role of the ub ligase FBXO7, in regulating mitophagy - highlighting that mutations in FBXO7 associate with Parkinson's disease and defects in mitochondrial homeostasis. Using the fly as model, they carry out a series of expts. investigating ntc (Drosophila ortholog of FBX07) demonstrating that it can functionally rescue Parkin but not PINK1 deficiency. Expanding on this, they propose a model whereby ntc/FBX07 regulates basal mitophagy and also acts as a priming Ub-ligase for Parkin mediated mitophagy, finding that the dub USP30 counteracts these ntc function. Overall the data are robust and support the authors' conclusions and model, the manuscript is well written and I think can be accepted as is.
- We thank the reviewer for their appreciation of the work and the time taken to provide supportive feedback.

While outside the scope of this study to understand why, I find it very interesting that ntc cannot rescue the PINK1 deficient phenotype, argues that PINK1 may be having additional effects beyond regulating mitochondrial ubiquitylation.
- We entirely agree with the reviewer, this is a very intriguing finding. Indeed, there are several examples in the literature showing that PINK1 performs additional functions than just triggering mitophagy. But in the current context we interpret these data as further support for a clear mechanistic distinction between basal mitophagy and stress-induced mitophagy as discussed at length to the other reviewers' comments.

Reviewer #2 (Significance (Required)):

Importance for understanding the role of FBX07 function - relevant for Parkinson's disease, also demonstrates a role for it in priming for PINK1/Parkin dependent mitophagy.

Reviewer #3 (Evidence, reproducibility and clarity (Required)):

In this manuscript, Sanchez-Martinez et al characterise the role of nutracker (ntc), the presumed Drosophila orthologue of human FBXO7 (whose gene is mutated in autosomal recessive PD), in

Review COMMONS

mitophagy and phenotypes associated with neurodegeneration in flies (climbing index, dopaminergic neuron loss, rough eye phenotype, and others). FBXO7 (human) has been previously shown to restore parkin (not Pink1) phenotypes and mitochondrial morphology in Drosophila and implicated in Pink1-parkin mitophagy, however the role of ntc in basal mitophagy and its genetic interaction with USP30 has not been previously reported. Key findings include: evidence for functional homology between ntc and FBXO7, and that Ntc/FBXO7 is required for basal mitophagy (and reverses USP30 function) in a Pink1-parkin independent manner.

FBXO7/ntc is clearly an important regulator of mitophagy and its overexpression can suppress Parkin phenotypes, however FBX07/ntc has not been studied as intensively as Parkin and Pink1, therefore this work represents important insight into mitophagy regulators (broad interest to many overlapping fields).

However, in addition to minor points and controls requested below, some further characterisation of the signals on the mitochondria induced by ntc/FBXO7 would improve the novelty of the study and the mechanistic insight provided. For example, the authors look at total ubiquitin and pS65-Ub, whereas if they looked at specific substrates that they mention in the discussion (e.g. OMM and translocon proteins) it would allow a less speculative discussion.

Figure 1:
The authors show that overexpression of ntc can rescue Parkin null phenotypes but not Pink1 phenotypes. In very similar experiments, overexpression of FBXO7 (human) has been shown to rescue Parkin phenotypes but not pink1 phenotypes (Burchell), appropriately mentioned by the authors.

The western blots are not terribly clear and would benefit from quantification (particularly H).
- We had previously performed the quantification on replicate experiments but had considered that the result was clear enough without quantification, and that including a quantification may make the figure too crowded. However, we have now added the quantification to the figure to support these results.

Specific ubiquitylated substrates like translocon proteins would be very interesting (alternatively, this could be provided in figure 7).
- We agree that this would be a very interesting aspect to investigate, but we feel that since the emphasis of the current study is clearly on the regulation of mitophagy, and not on specific substrates as has been published elsewhere (Phu et al., Mol Cell, 2020 (Ref. 42); Ordureau et al., Mol Cell, 2020 (Ref. 39)), investigating the impact of ntc and USP30 on the ubiquitination of the translocon would be a distraction to the focus of the study.

If the rough eye phenotype is highly homogenous, state in text otherwise, a relative roughness quantification would be more informative.
- The rough eye phenotype described here is indeed highly stereotyped and homogeneous. We have added this comment to the text for clarity (lines 124-126).

Figure 2:
Although mainly in agreement with Burchell et al findings (that there is no disruption of mito morphology or dopaminergic neuron loss caused by ntc loss), the loss flight ability in the ntc mutant is partially discrepant with Burchell et al (results not shown in Burchell et al). Can the authors explain the discrepancy? It is important finding that the ntc functionally orthogue of FBXO7 and differs from the Burchell et al conclusions.

- The reviewer raises a good point regarding discrepant interpretations with earlier preliminary work that we didn't specifically elaborate in the current manuscript. For the Burchell et al study we performed a series of non-exhaustive analyses with reagents that were most readily available at the time. The flight data described in Burchell et al (as 'data not shown') were done with what we now know to be a hypomorphic allele, which did not give a strong flight defect that we were expecting to see as a phenocopy of *parkin* mutants. Moreover, experiments aimed at testing the functional homology sought to rescue the only reported *ntc* phenotype at that time – male sterility – which did not work. It is worth noting that GAL4/UAS-mediated expression is known to be very inefficient in the male germline, so we originally interpreted the lack of rescue with this caveat in mind. It is also worth adding that, subsequent to the Burchell et al study, we have seen that expression of FBXO7 can rescue the caspase-3 activation in *ntc* mutant spermatocytes, supporting their functional homology. Importantly, during the Burchell et al study we did not have reagents to test the effects of *ntc* overexpression, obtained subsequently, which have provided compelling data that support a functional homology between ntc and FBXO7. At the time of writing the current manuscript we did not specifically revisit the Burchell et al text to note this strongly stated conclusion. We realise that this requires unequivocal clarification and thank the reviewer for pointing this out. We have amended the text to clarify this important point (lines 285-293).

Figure 7: A,B. It is not clear that mitochondria have been enriched - can the authors show on mitochondria or show the fractionation quality?

- Mitochondrial enrichment is a standard procedure in our lab, with consistently acceptable results, so we apologize for omitting a demonstration of this. We have now added these data to a new supplementary figure S5A. The corresponding information has also been added to the text (line 253). We have also extended this analysis to now show that total ubiquitination is not changed in *ntc* OE or *USP30* RNAi, highlighting the specificity for accumulated ubiquitination on the mitochondria. This has been added to supplementary figure S5Band text lines 253-254.

C/D. The text that accompanies these figures needs further explanation and clarification and I found this result hard to understand without referring to the discussion. I think the authors are concluding that pS65 is ubiquitylated by FBXO7? I think this should be re-written in the results section. If it is a major point that the authors want to make, a complementary approach would be advised - possibly human cells/mass spectrometry.

- We apologise that this was confusing and have simplified the text accordingly to improve the clarity (lines 260-262). While this specific analysis is not a major point of the study, it provides a useful additional measure of how ntc/USP30 contributes to mitochondrial ubiquitination which *is*

Review COMMONS

a key focus of the study so we have revised the Discussion to better highlight this point (lines 359-387).

As for Figure 1, specific ubiquitylated substrates at the OMM such as the translocon subunits would be informative.
- As discussed above, the role of USP30, at least, on ubiquitination of protein import in the translocon has been documented elsewhere and further specific analysis on this here would be a distraction from the main focus of the study.

Minor points

Figure 8 model and discussion:
Nice discussion. However, unless protein import/ubiquitylation of translocon factors/localisation of FBXO7 to the translocon is shown in the manuscript, I would recommend more clarity in the figure legend to emphasise what is speculation based on other papers and what are new findings from the paper.
- This is a fair point and we agree that it is good to be clear about which aspects of the working model are reflections of the data presented here and which are extrapolation/speculation from the literature. We have modified the figure and the figure legend accordingly.

Reviewer #3 (Significance (Required)):

FBXO7/ntc is clearly an important regulator of mitophagy however its mechanism of action has not been studied as intensively as Parkin and Pink1, therefore this work contains important insight into mitophagy regulators.

It will be of broad interest to many overlapping fields, and has translational impact in that mitophagy is disrupted in many diseases and FBXO7 itself is mutated in Parkinson's disease.
